# Professionalism vs. engagement: quality of SSc information on WeChat

**DOI:** 10.3389/fpubh.2025.1527853

**Published:** 2025-05-14

**Authors:** Lei Wang, Yue Xiong, Tingting Wu, Yingying Gao, Haojie Chen, Xin Chu, Baofeng Zhu, Jing Cao, Tao Cheng, Mingjun Wang

**Affiliations:** ^1^Department of Rheumatology and Immunology, The First Affiliated Hospital of Soochow University, Suzhou, China; ^2^Department of Emergency Medicine, The First People’s Hospital of Nantong, Nantong, China; ^3^Department of Rheumatology and Immunology, The First People’s Hospital of Nantong, Nantong, China

**Keywords:** systemic sclerosis, rare diseases, autoimmune disease, WeChat public account, health information quality, DISCERN, social media

## Abstract

**Background:**

Systemic sclerosis (SSc) is a rare autoimmune disease, and WeChat is a major source of health information in China. This study assesses the quality of SSc information on WeChat to understand its impact on public knowledge and engagement.

**Methods:**

A total of 375 articles from 9 WeChat public accounts were systematically analyzed using the DISCERN and Global Quality Scale (GQS) tools. Article quality was evaluated based on source credibility, content accuracy, and user engagement, including metrics such as views, likes, and comments.

**Results:**

Individual authors posted 50% of the articles, while non-profit organizations posted 21%, with non-profits providing higher quality content. Disease knowledge dominated (52.8%), yet readers showed higher interest in policy interpretation and rehabilitation. The average DISCERN and GQS scores were 28.96 and 1.62, indicating low quality across articles.

**Conclusion:**

While WeChat facilitates SSc information dissemination, the overall quality is lacking. Enhancing professionalism and interactivity on health information platforms like WeChat could better meet the needs of patients and the public for reliable information.

## Introduction

Systemic sclerosis (SSc), also known as scleroderma, is a rare and complex autoimmune disease characterized by fibrosis of the skin and internal organs, vasculopathy, and immune dysregulation (1–4). Its pathogenesis remains incompletely understood, involving genetic susceptibility, environmental triggers, and immune abnormalities. SSc is more prevalent in North America and Europe, with a U.S. prevalence of approximately 50 per 100,000 and an incidence of 5.6 per 100,000 person-years, predominantly affecting women aged 30–50 (5, 6). The disease often leads to widespread vascular dysfunction and multi-organ involvement, with prognosis strongly influenced by disease subtype (diffuse vs. limited) and the presence of pulmonary arterial hypertension (PAH) or interstitial lung disease (ILD) (7, 8). Although epidemiological data are limited in China, incidence appears to be rising, with significant diagnostic and treatment challenges in resource-limited areas.

In modern society, access to reliable health information is crucial for patients and their families to manage diseases effectively (9, 10). With the rapid development of the internet and information technology, social media has become an important source of health-related content (11, 12). These platforms provide convenient access to information and facilitate communication among patients, contributing to the formation of broader support networks (13, 14). In China, WeChat serves as the most widely used social media platform, offering distinct advantages for health communication due to its large user base and multifunctionality (15, 16). Through official accounts, medical institutions, non-profit organizations, and individuals can share information on disease knowledge, treatment approaches, and recent medical developments (17–19). Ensuring the scientific rigor and reliability of this information remains essential for its effective dissemination (20–22).

The widespread use of WeChat official accounts presents new opportunities for disseminating rare disease information (23). With features such as real-time updates, strong interactivity, and broad reach, WeChat has become an important tool for health communication. However, the quality of health information on the platform varies, with some content lacking scientific basis or potentially misleading. It affects patient decision-making and health management and impacts the credibility of information providers and the platform itself (24–26). Studies on health communication via WeChat show that users can engage through comments, likes, and shares, enhancing the spread and understanding of health content (27). The platform’s real-time update capability also helps patients and families access the latest health information and supports personalized health management (28, 29).

Assessing the quality of health information is essential for ensuring that patients receive reliable content (30, 31). DISCERN and Global Quality Scale (GQS) are commonly used tools for evaluating health content’s scientific accuracy, practicality, and credibility (32–34). DISCERN focuses on information reliability and treatment options quality, while GQS assesses overall quality and usability (35–37). Applying these tools helps ensure patients receive evidence-based guidance, enhances the credibility of health information, and improves communication effectiveness (38–40). Access to high-quality information is particularly important in rare diseases such as SSc. As a complex autoimmune disorder with diverse clinical manifestations and treatment strategies, SSc requires patients and healthcare providers to rely on accurate medical information for effective management and improved quality of life. Enhancing information quality and optimizing health communication through social media can positively contribute to public health education and patient care.

The study evaluates the quality of treatment information for systemic sclerosis on WeChat and analyzes the long-term operational factors of related WeChat public accounts in information dissemination. Identifying gaps in information quality encourages health information providers to enhance professionalism and reliability, improving patients’ access to accurate information. The findings also inform rare disease communication strategies and promote the establishment of more effective information-sharing mechanisms on WeChat by healthcare institutions and non-profit organizations.

## Materials and methods

### Ethical considerations

Ethical considerations the information utilized in this study was solely derived from publicly available WeChat data, with no involvement of any personal privacy concerns. Clinical data or human specimens were not included in the research, nor was there any direct interaction with users. Consequently, ethical review was deemed unnecessary.

### Data collection and analysis

On September 10, 2023, data was collected from WeChat, Baidu, Qingbo, and Sogou, using the keywords “Systemic Sclerosis” or “scleroderma” to identify relevant Chinese WeChat public accounts. Due to the limited number of SSc-related accounts, manual data collection was employed to ensure quality. Researchers manually searched and recorded details of public accounts related to systemic sclerosis or scleroderma, excluding deactivated accounts, those inactive for over 6 months, and accounts not focused on SSc. Account details were documented, such as registration date, operator, posting frequency, and article count.

**Figure 1:**
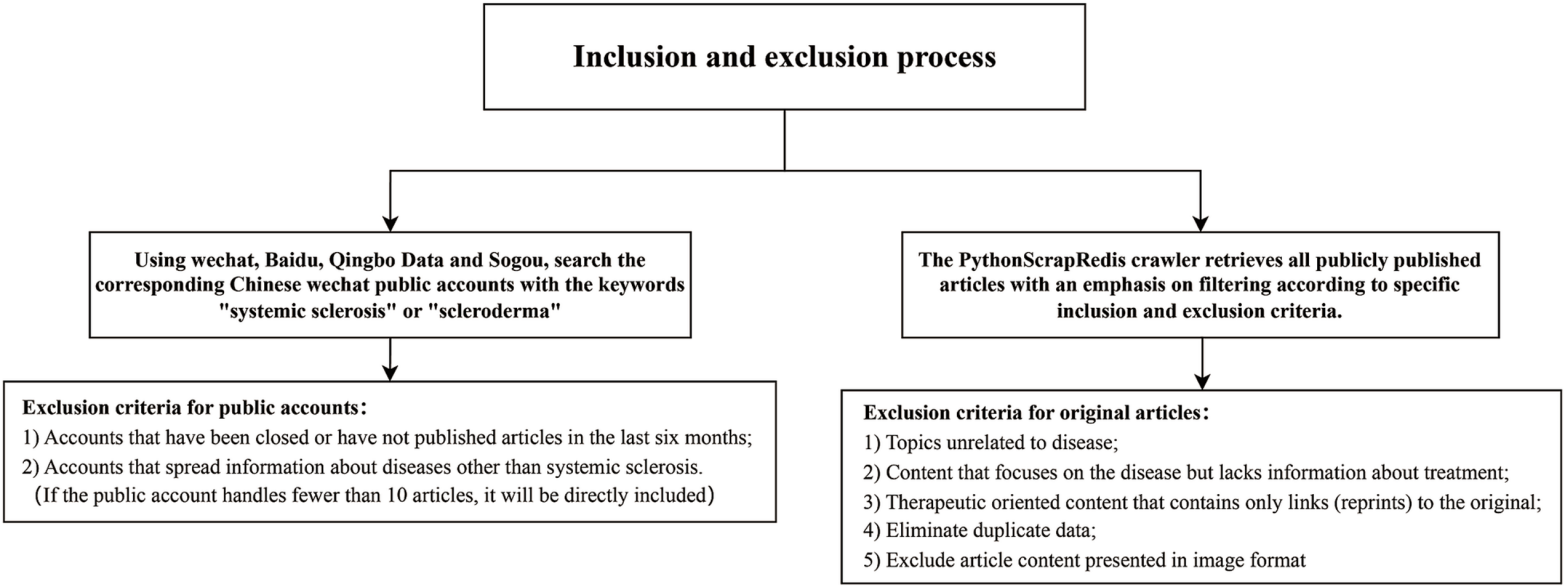
Data inclusion and exclusion flowchart.

Using the PythonScrapRedis crawler, all publicly published articles were retrieved and filtered according to specific inclusion and exclusion criteria. PythonScrapRedis, combining Python and Redis, facilitated efficient automated data scraping and processing. Inclusion criteria required articles to be original and provide treatment information related to rare diseases (RDs), while exclusion criteria eliminated irrelevant topics, content without treatment details, reposted links, duplicates, and articles in image format unsuitable for DISCERN evaluation (Figure 1).

This research was designed as a cross-sectional study, with sample size calculation based on previous studies (41–43). An average DISCERN score of 32.3 (SD = 11) was expected. With a 5% invalid data rate, a minimum of 218 cases was required. Proportional random sampling was conducted using R3.4.2 software, ensuring representativeness. Accounts with fewer than 10 processed articles were fully included to maintain sampling accuracy.

### Classification of articles

Articles are categorized based on the primary source of WeChat operations (how different entities or organizations publish and manage content on the platform) and the treatment methods introduced in each article. The sources of articles are divided into three categories: (1) public interest organizations (medical funds), (2) commercial companies, and (3) individuals/patients without medical professional backgrounds. The content categories are as follows: (1) functional rehabilitation; (2) nursing; (3) disease knowledge; (4) Western medicine; (5) psychology; (6) policy interpretation; (7) traditional Chinese medicine; and (8) comprehensive treatment involving two or more modalities. The articles are downloaded, and information extracted between September 10, 2023, and October 10, 2023.

### Evaluation tools

This study assessed the quality of health information using the DISCERN tool and the GQS. DISCERN evaluates treatment-related health information through 16 items divided into three sections: Part 1 (questions 1–8) assesses reliability, Part 2 (questions 9–15) focuses on treatment quality, and Part 3 (question 16) provides an overall rating (43, 44). Each item is scored on a 5-point scale based on five key criteria: clarity and achievement of objectives, use of reliable sources such as published literature or expert opinion, balance and impartiality of content, availability of additional resources, and discussion of uncertainties. Total scores range from 16 to 80 and are classified as very poor (16–26), poor (27–38), fair (39–50), good (51–62), and excellent (63–80).

The GQS provides an overall assessment of patient content quality and educational value (43, 45). A score of 1 indicates poor quality, missing information, or misleading content with minimal educational value; 2 indicates limited and incomplete content with low technical quality; 3 reflects moderate quality, partially informative content, and basic technical adequacy; 4 indicates good quality with comprehensive and useful content; and 5 represents excellent quality, complete information, and strong educational value.

All evaluations were independently conducted by two senior rheumatologists with extensive clinical experience in systemic sclerosis (Haojie Chen and Yingying Gao). Each rater applied DISCERN and GQS to assess the selected content, with final scores calculated as the average. In cases of significant discrepancy, consensus was reached through team discussion. Inter-rater reliability was assessed using Pearson correlation analysis based on raw scores.

### User feedback and behavioral analysis

Two individuals recorded the data. The researchers were experienced senior rheumatologists proficient in diagnosing and treating SSc, utilizing the DISC method during the screening and grading process.

This study delves into understanding user satisfaction and concerns by collecting user feedback on SSc-related articles on the WeChat platform. The specific methods involved gathering user comments and questions and classifying and conducting sentiment analysis on these responses using natural language processing techniques. User comments and feedback were classified using a Naive Bayes classifier into positive, neutral, and negative categories. Sentiment analysis models and modern pre-trained models (e.g., BERT, GPT) were employed to capture and interpret emotional content in the comments. The study assessed post-article behaviors by analyzing user engagement data, including shares, discussions, and click-through rates. Data collection was facilitated through the data interfaces provided by the WeChat public platform, enabling user comments, shares, and click data extraction.

### Application of multiple assessment tools

For a comprehensive and accurate evaluation of therapeutic information related to SSc on the WeChat platform, this study introduced various tools for assessing the quality of medical information. Initially, the accuracy and timeliness of medical information were assessed, evaluating the authenticity, reliability, and whether the content reflects the latest research or clinical guidelines. Rheumatology experts scored the articles to ensure content accuracy and relevance. A reader satisfaction questionnaire was also designed and distributed to collect readers’ evaluations of the articles’ content, format, and practicality through the WeChat public platform. The questionnaire results were compared and analyzed against the scores from other assessment tools to provide a comprehensive quality assessment. Finally, the survey results were combined with other assessment tools for a comprehensive quality evaluation. Finally, the study developed an integrated evaluation framework using DISCERN and GQS scores to rank and assess the overall quality of the articles.

### Statistical methods

Continuous variables were presented using the mean and standard deviation, while non-continuous variables were depicted using the median (interquartile range, IQR). Percentages represented categorical variables. Group differences were assessed using the Kruskal-Wallis test for non-normally distributed quantitative variables and Dunn’s multiple comparison test for pairwise comparisons. Spearman correlation analysis was employed to evaluate relationships between quantitative variables. Statistical significance was defined as *p* < 0.05. Statistical analyses were performed using GraphPad Prism 9.0.0 for Windows (GraphPad Software).

## Results

### Key directions to enhance content quality and user engagement

This study analyzed 6,408 articles from 9 WeChat public accounts, of which 59% were original content. A random sample of 375 articles was further examined (Table 1). The analysis showed high readership for SSc-related articles, with a total of 172,841 views and a median of 265 views per article. However, user interactions (likes, comments, and shares) were relatively low, with only 2,094 likes, 705 comments, and 1,377 shares. This suggests that despite significant interest in the topic, user engagement remains limited, indicating a one-way flow of information.

**Table 1:** General information of the public number.

Serial number	Official account	Registration time	Operate body	Last watch interval (days)	Post frequency (week/post)	Number of posts	Number of posts read (M)	Original (%)
1	“Scleroderma Terminus”	20,190,703	Personal	7	3	210	60	208 (99)
2	“Multiple Sclerosis Home”	20,200,326	Institution	1	3	690	693.5	130 (18)
3	“Multiple Sclerosis Research and Treatment Center”	20,200,423	Personal	24	1	147	501	85 (58)
4	“Inner Mongolia Systemic Sclerosis Home”	20,170,921	Personal	19	2	199	23	37 (19)
5	“Scleroderma Systemic Sclerosis”	20,191,031	Individual	1	32	2,765	2	1,682 (61)
6	“Demyelination Fragmentation Mindset”	20,170,101	Personal	51	8	655	262	224 (34)
7	“Science popularization of scleroderma”	20,230,715	Personal	1	4	40	2	40 (100)
8	“Purple Conch Public Welfare Service Center”	20,200,707	Institution	2	7	1,007	422	535 (53)
9	“Scleroderma”	20,160,204	Company	62	2	695	185	690 (99)

*Last correction interval (days): indicates the time difference between the search date and the date the article was last updated.

Among the accounts, “Multiple Sclerosis Home” showed high activity and readership but relied heavily on reposted content, with a low proportion of original articles. On the other hand, “Scleroderma Systemic Sclerosis” had high posting frequency but low engagement, possibly due to content quality or distribution issues. Notably, “Purple Conch Public Welfare Service Center” and “Scleroderma” demonstrated strong original content creation and good user interaction, highlighting their effective content strategies.

### The increasing role of WeChat platform in SSc information dissemination

**Figure 2:**
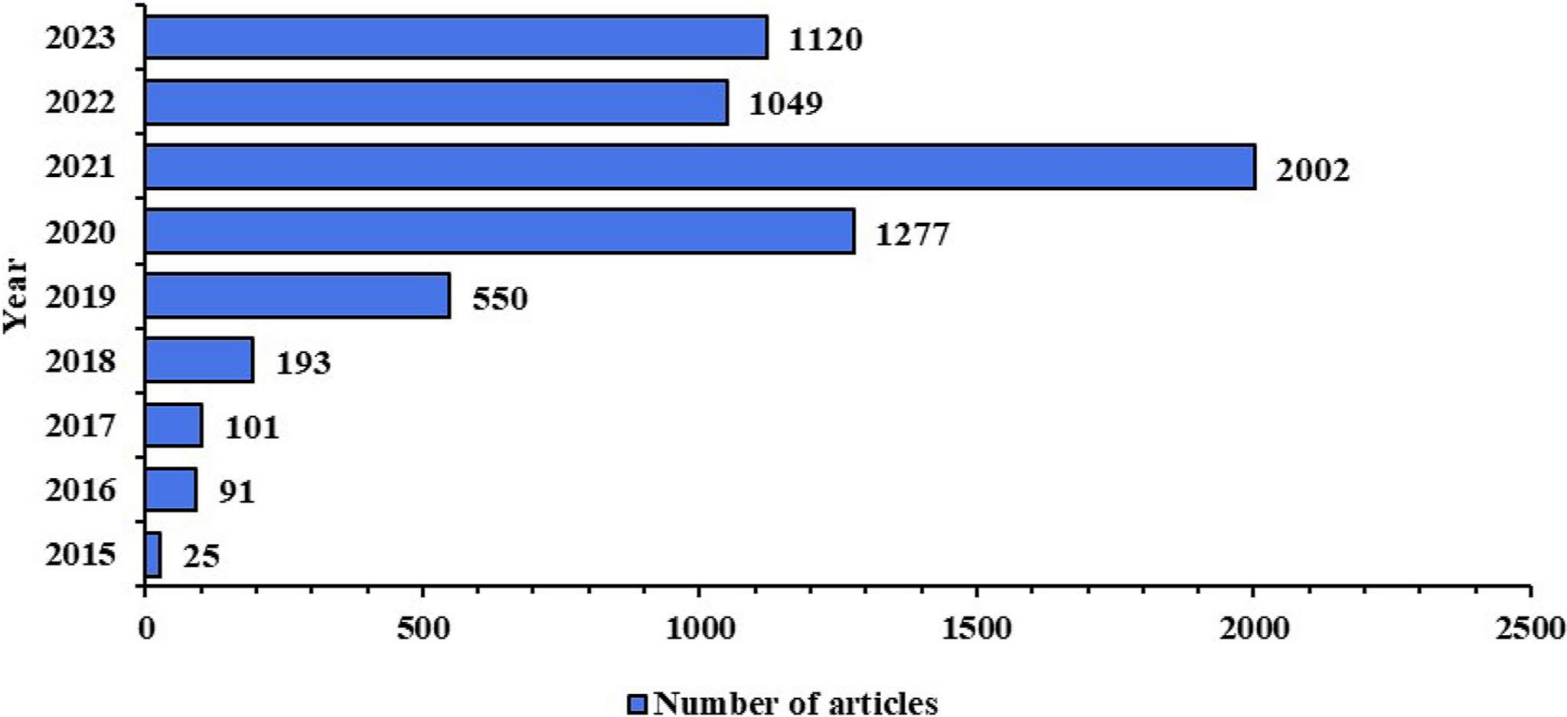
Distribution pattern of publication time in the academic paper.

This study analyzed the time distribution of 6,408 articles published between January 1, 2015, and September 10, 2023, to monitor the activity of WeChat public accounts. Starting in 2019, publication frequency increased noticeably, with a significant peak observed during 2020–2021 (Figure 2).

The data shows that in 2015 and 2016, the number of articles was relatively low, with 25 and 91 articles, respectively. In 2017 and 2018, the number increased slightly to 101 and 193 articles, indicating modest growth. A significant rise occurred in 2019, with 550 articles published, marking increased activity in public accounts. In 2020 and 2021, articles surged to 1,277 and 2,002, respectively. This spike can be attributed to the expansion of the WeChat user base and a growing demand for SSc-related information. Although the number of articles slightly decreased in 2022 and 2023 to 1,049 and 1,120, respectively, they remained relatively high.

### Optimizing content strategy to enhance user engagement

**Figure 3:**
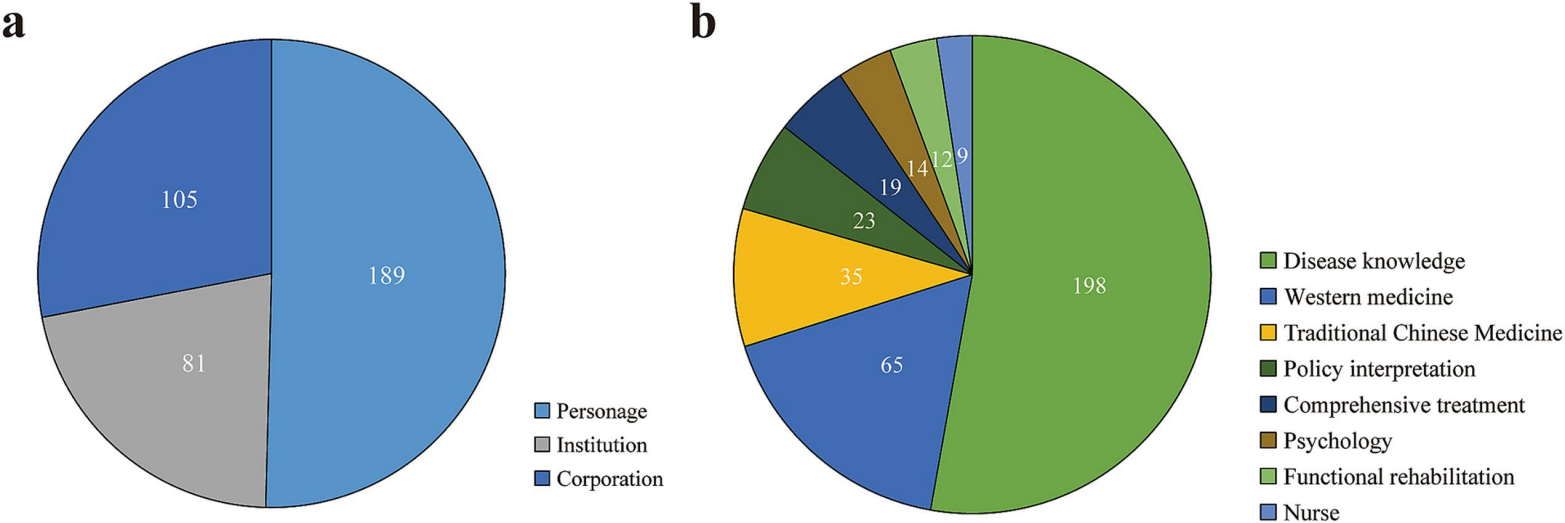
Analysis of article sources and content characteristics. **(a)** Distribution of article sources from the SSc topic on the WeChat public account. **(b)** Distribution of article themes from the SSc topic on the WeChat public account.

Based on the cited sources (Figure 3a), individuals account for 50% of the articles (189/375), followed by companies at 28% (105/375) and institutions at 21% (81/375). In terms of content, disease knowledge dominates, representing 53% of the total articles (198/375), while other content types include Western medicine (17%), traditional Chinese medicine (9%), and policy interpretation (6%) (Figure 3b). Table 2 presents descriptive statistics for these sources and content types.

**Table 2:** Characteristics of articles by sources and contents [M(IQR)].

Variables	*N*%	Reading	Likes	Comments	Watching
Article sources					
Personal	189 (50.4)	110 (41,481)	4 (0,8)	0 (0,0)	4 (1,8)
Institution	81 (21.6)	538 (342,1,012)	2 (1,6)	0 (0,1)	1 (0,3)
Company	105 (28)	265 (168,356)	1 (0,2)	1 (0,3)	1 (0,1)
Article content					
Functional Rehabilitation	12 (3.20)	852 (68,1,078)	6 (1,13)	0 (0,0)	7 (1,12)
Nursing Matters	9 (2.40)	146 (39,217)	2 (0,4)	0 (0,4)	1 (1,3)
Disease Knowledge	198 (52.8)	217 (82,398)	2 (0,4)	0 (0,1)	1 (0,3)
Western Medicine	65 (17.33)	450 (310,848)	2 (1,5)	0 (0,1)	1 (0,3)
Mental	14 (3.73)	300 (185,694)	7 (5,16)	1 (0,8)	5 (3,9)
Policy Interpretation	23 (6.13)	1,035 (523,1,379)	22 (6,35)	4 (1,8)	10 (5,16)
Traditional Chinese Medicine (TCM)	35 (9.33)	81 (32,167)	2 (0,5)	0 (0,0)	2 (0,4)
Combination Treatment	19 (5.07)	100 (59,937)	6 (1,13)	0 (0,0)	4 (1,11)

Articles on policy interpretation (1,035 views) and functional rehabilitation (852 views) garnered the highest readership, indicating strong reader interest. Articles from institutions had an average reading volume of 538, significantly higher than those from individuals (110) and companies (265), suggesting greater reader engagement with authoritative sources.

Despite the many disease knowledge articles, their user engagement and readership were relatively low. Institutional articles had the highest readership but fewer likes and comments. Although individuals published the most articles, their engagement and readership were moderate. Company articles ranked between those from individuals and institutions across all metrics. Policy interpretation articles had the highest user interaction, followed by functional rehabilitation. Articles on Western medicine and psychology showed moderate readership but lower interaction, while traditional Chinese medicine and comprehensive therapy had the lowest engagement. In summary, policy interpretation and functional rehabilitation articles attracted more readership and engagement than other content types.

### The urgency of enhancing information quality on SSc through DISCERN evaluation

**Figure 4:**
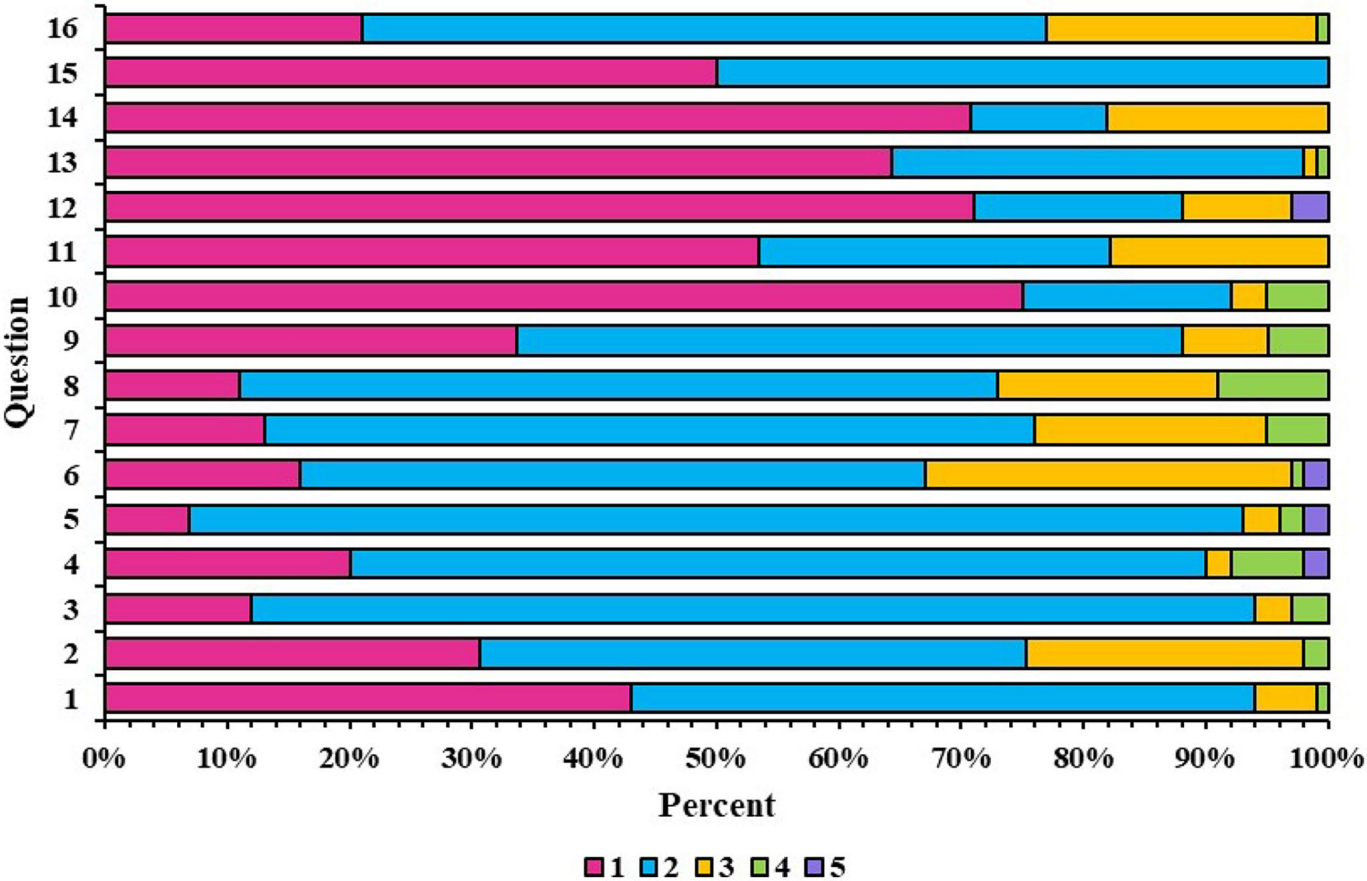
Individual distribution of DISCERN scores in percentage.

This study assessed the quality of systemic sclerosis-related articles on WeChat using the DISCERN tool (Table 3). The results showed an average score of 28.96 (SD = 7.13), with an overall quality rating of “poor.” The analysis indicated that scores were mainly concentrated in Parts 1 and 2, with few high scores (4 and 5), reflecting deficiencies in content reliability and treatment details (Figure 4).

**Table 3:** Article DISCERN score by item ( $\bar{X}$ ± S).

Questions (1–5 marks/item)	$\bar{X}$ ± S	95%CI
Reliability of article content (8 items)		
1. Is the goal clear?	1.64 ± 0.63	1.57 ~ 1.69
2. Have you achieved your expected goals?	1.96 ± 0.78	1.87 ~ 2.03
3. Does the content meet the needs of patients?	1.97 ± 0.51	1.91 ~ 2.01
4. Is the source of the content information clear?	1.98 ± 0.78	1.91 ~ 2.07
5. Is the source of the information used or reported in the article clear?	2.04 ± 0.57	1.98 ~ 2.10
6. Is it objective and impartial?	2.22 ± 0.79	2.14 ~ 2.30
7. Do you provide details such as sponsor and citation information?	2.17 ± 0.70	2.09 ~ 2.23
8. Does it mention areas that have not yet been defined?	2.25 ± 0.77	2.17 ~ 2.32
Details of treatment information (7 items)		
9. Is each treatment option described?	1.83 ± 0.76	1.76 ~ 1.91
10. Are the benefits of each therapy described?	1.38 ± 0.78	1.3 ~ 1.46
11. Are the risks of each therapy described?	1.64 ± 0.76	1.57 ~ 1.72
12. Does it describe the possible consequences of not pursuing treatment?	1.48 ± 0.9	1.39 ~ 1.57
13. Is the impact of treatment options on quality of life described?	1.37 ± 1.31	1.31 ~ 1.42
14. Is there a clear description of the multiple therapies that may exist?	1.47 ± 0.78	1.39 ~ 1.56
15. Do you support joint decision making?	1.50 ± 0.5	1.45 ~ 1.56
Overall article quality (1 item)		
16. Based on the above questions, score the overall quality	2.04 ± 0.69	1.96 ~ 2.11
Total points	28.96 ± 7.13	28.24 ~ 29.69

In Part 1 (content reliability), the average score for 8 items was 16.23 (SD = 3.42). Better-performing questions included “Is the information objective and impartial?” (2.22, SD = 0.79) and “Does it address undefined areas?” (2.25, SD = 0.77). However, low scores for “Is the objective clear?” (1.64, SD = 0.63) and “Has the objective been achieved?” (1.96, SD = 0.78) indicated weaknesses in goal setting and achievement.

In Part 2 (treatment information details), the average score for 7 items was 10.69 (SD = 3.75). The lowest scores were for “Does it describe the benefits of each therapy?” (1.38, SD = 0.78) and “Does it describe the impact of treatment options on quality of life?” (1.37, SD = 1.31), indicating significant information gaps. Relatively higher scores were observed for “Does it describe each treatment option?” (1.83, SD = 0.76) and “Does it describe the risks of each therapy?” (1.64, SD = 0.76), but overall, these aspects were still lacking.

In Part 3 (overall article quality), the average score was 2.04 (SD = 0.69), indicating poor overall quality, particularly due to the lack of detailed descriptions of treatment benefits and quality of life impacts.

### DISCERN scores for different categories

**Figure 5:**
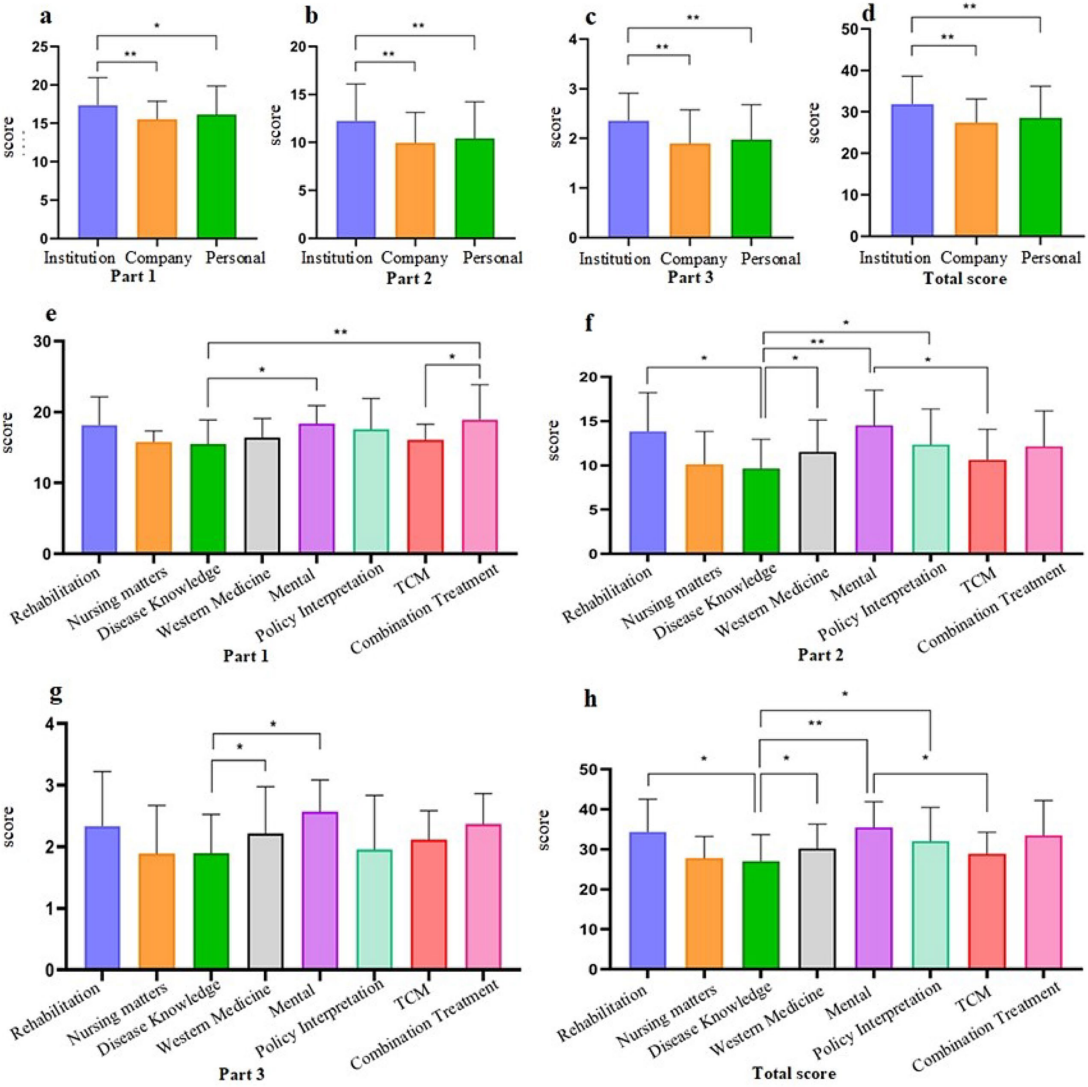
DISCERN scores originating from different sources **(a–d)** and content types **(e–h)** (*p < 0.05, **p < 0.001).

The DISCERN scores for articles from different sources (Figures 5a–d) and content types (Figures 5e–h) were evaluated (Figure 5). In Part 1 (Content Reliability), non-profit organizations scored significantly higher than businesses and individuals (*p* = 0.0007 and *p* = 0.016). Articles on common knowledge and psychology outperformed those on traditional Chinese medicine and disease knowledge (*p* = 0.046, *p* = 0.0005, and *p* = 0.042), highlighting the higher reliability of non-profit articles, particularly in these areas.

In Part 2 (Treatment Information Details), non-profit organizations again achieved significantly higher scores than businesses and individuals (*p* < 0.0001 and *p* = 0.0005). Articles on functional rehabilitation, Western medicine, and policy interpretation scored better than those on disease knowledge (*p* = 0.002, *p* = 0.0058, and *p* = 0.014), while psychology also outperformed both disease knowledge and traditional Chinese medicine (*p* < 0.001 and *p* = 0.013).

In Part 3 (Overall Quality), non-profit organizations continued to lead with significantly higher scores compared to businesses and individuals (*p* < 0.0001 in both comparisons). Articles on psychology and Western medicine scored better than disease knowledge (*p* = 0.006 and *p* = 0.017). Overall, non-profit organizations had superior total scores across all sections (*p* < 0.0001 and *p* = 0.009), with articles on functional rehabilitation, Western medicine, policy interpretation, and psychology consistently outscoring disease knowledge and traditional Chinese medicine (*p* = 0.007, *p* = 0.023, *p* = 0.019, *p* = 0.0002, and *p* = 0.043).

### GQS scores for articles of different categories

**Figure 6:**
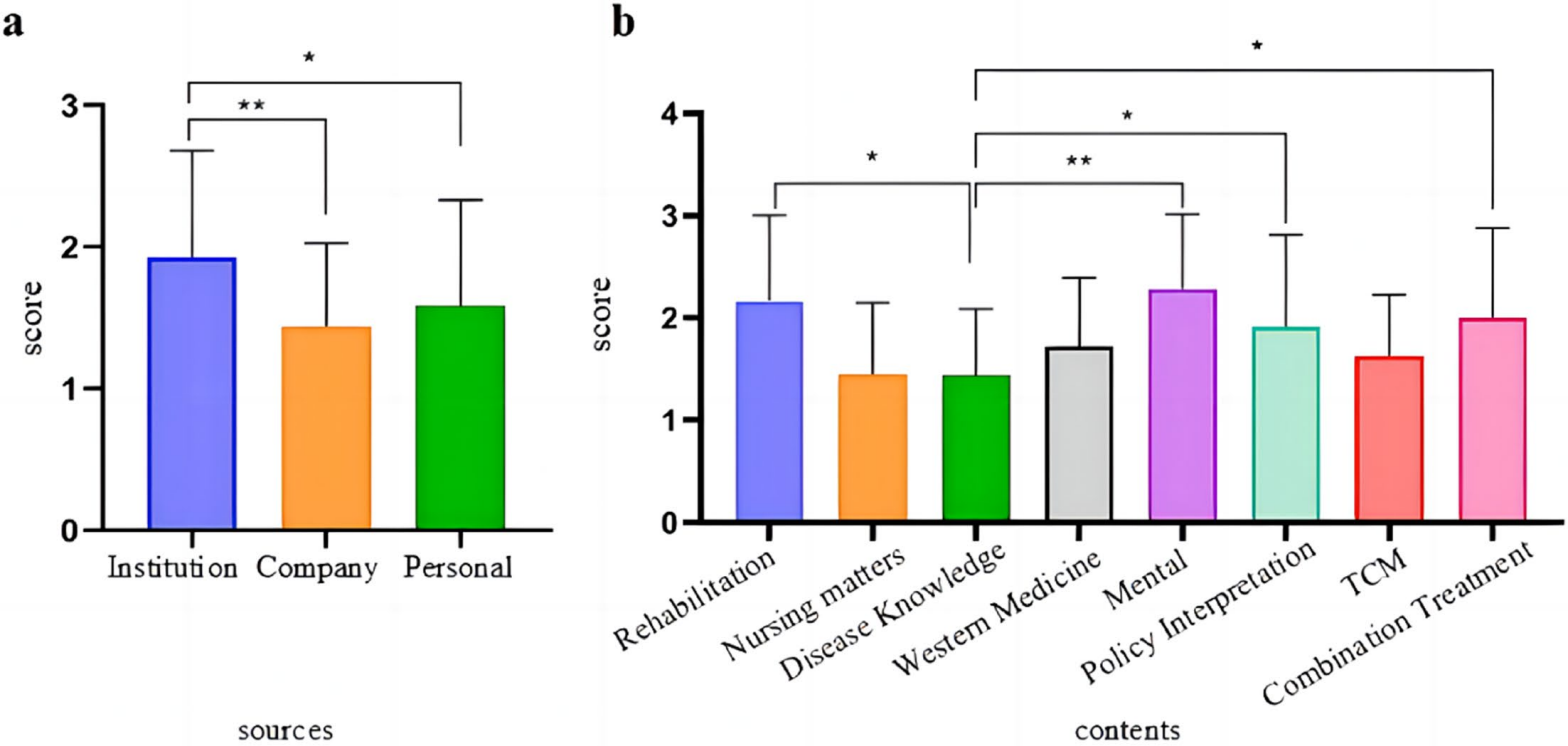
Global Quality Scale (GQS) scores from various sources **(a)** and contents **(b)** (*p < 0.05, **p < 0.001).

Based on the evaluation of article quality from different sources (a) and content (b), the overall average GQS score was 1.62 (SD = 0.72), indicating a classification of “poor.” The results depicted in Figure 6 show that the scores of articles from public organizations were significantly higher than those from companies and individuals (*p* < 0.0001 and *p* = 0.001). Functional rehabilitation, psychology, policy analysis, and general knowledge scores were notably higher than disease knowledge (with respective *p*-values of 0.01, 0.000, 0.04, and 0.017). The findings indicate that the quality of articles from public organizations surpasses those from businesses and individuals. Additionally, articles relating to functional rehabilitation, Western medicine, psychology, policy analysis, and general knowledge demonstrated higher quality than others.

**Figure 7:**
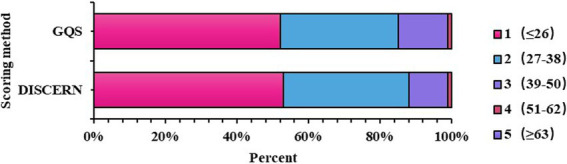
Distribution of Scores for DISCERN and GQS.

Based on the DISCERN and GQS scores mentioned above, it was found that the quality of WeChat articles related to SSc was subpar, with a maximum DISCERN score of 199 (375 points, accounting for 53%) and a maximum GQS score of 196 (100 points, accounting for 52%). Upon pairwise comparison of the five levels in DISCERN and GQS, no significant differences were observed (*Z* = −0.105, *p* = 0.916), indicating that both scoring systems used for evaluation equally reflected article quality (Table 4 and Figure 7).

**Table 4:** Five-level distribution of DISCERN and GQS (*n* = 375).

Variables	*N*%
DISCERN	
≤26, Very bad	199 (53)
27–38, Poor, average	132 (35)
39–50, Intermediate	43 (11)
51–62, Good	1 (1)
≥63, Excellent	0 (0)
GQS	
1 Very poor	196 (52)
2 Poor, average	127 (33)
3 Medium	51 (14)
4 Good	1 (1)
5 Excellent	0 (0)

### Correlation analysis: enhancing article quality to increase user engagement

Spearman correlation analysis reveals significant relationships among the number of reads, likes, comments, and views of articles on SSc. Specifically, a strong positive correlation is observed between the number of reads and likes (*r* = 0.54, *p* < 0.0001), indicating that articles with higher readership tend to receive more likes. Furthermore, a moderate positive correlation exists between reads and views (*r* = 0.42, *p* < 0.0001), suggesting increased readership is associated with more views. While the correlation between comments and reads is weak (*r* = 0.11, *p* = 0.042), it is statistically significant. There is also a moderate positive correlation between likes and comments (*r* = 0.16, *p* = 0.003) and a strong correlation between likes and views (*r* = 0.55, *p* < 0.0001). Overall, there is a strong positive correlation between comments and views (*r* = 0.33, *p* < 0.0001), indicating a certain interplay of user interaction across different metrics (Table 5).

**Table 5:** Correlation analysis among variables.

Variables	Statistics	Reads	Likes	Comments	Watching
Reads	*R*-value	1	–	–	–
	*p*-value	–	–	–	–
Likes	*R*-value	0.54	1	–	–
	*p*-value	< 0.0001^b^	–	–	–
Comments	*R*-value	0.11	0.16	1	–
	*p*-value	0.042^a^	0.003^a^	–	–
Watching	*R*-value	0.42	0.55	0.33	1
	*p*-value	< 0.0001^b^	< 0.0001^b^	< 0.0001^b^	–

^a^*P < 0.05, ^b^**p < 0.001.

Furthermore, the study indicates a significant positive correlation between article quality scores (DISCERN score) and the number of reads (*r* = 0.37, *p* < 0.0001), likes (*r* = 0.33, *p* < 0.0001), and views (*r* = 0.28, *p* < 0.0001). GQS score also shows a positive correlation with reads (*r* = 0.35, *p* < 0.0001), likes (*r* = 0.29, *p* < 0.0001), and views (*r* = 0.26, *p* < 0.0001). These findings suggest that articles of higher quality are more likely to attract readers, likes, and views (Table 6).

**Table 6:** Correlation analysis between variables and ratings.

Variables	Statistics	DISCERN	GQS
Reads	*R*-value	0.37	0.35
	*p*-value	<0.0001	<0.0001
Likes	*R*-value	0.33	0.29
	*p*-value	<0.0001	<0.0001
Comments	*r*-value	0.09	0.08
	*p*-value	0.05	0.09
Watching	*R*-value	0.28	0.26
	*p*-value	<0.0001	<0.0001

^a^*P < 0.05, ^b^**P < 0.001.

### User feedback and behaviors revealing information needs

**Figure 8:**
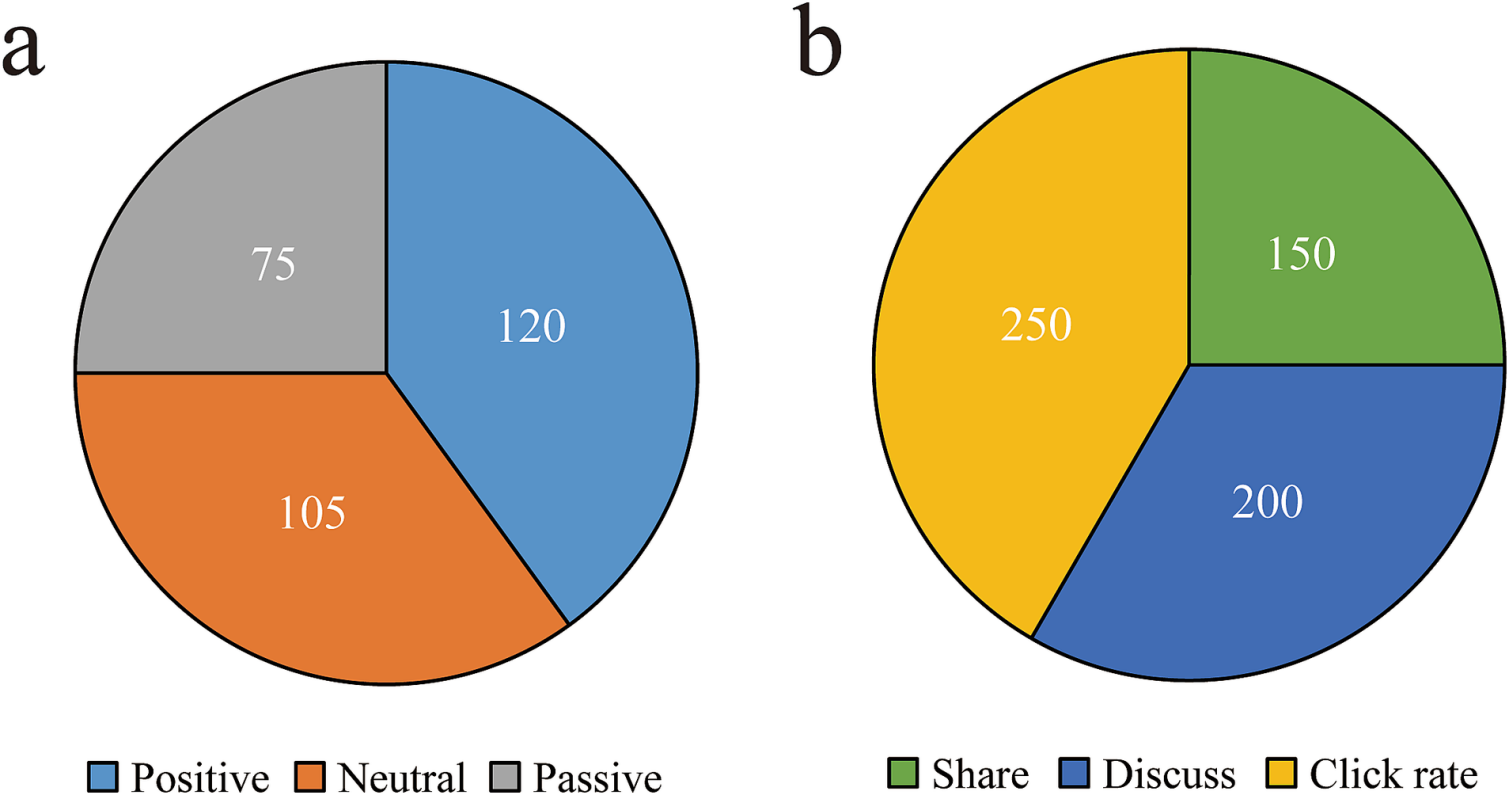
Analysis of user comments and behavior data. **(a)** Results of sentiment analysis of user comments. **(b)** Results of analysis of user behavior data.

The sentiment analysis of user comments reveals that out of a total of 300 comments, positive sentiments hold the highest proportion at 40% (120 comments), followed by neutral comments at 35% (105 comments) and negative comments at 25% (75 comments) (Table 7 and Figure 8A). Furthermore, the study examines user behavior data following article publication, encompassing sharing, discussing, and click-through rates. The analysis of user behavior data indicates that post-reading actions primarily concentrate on click-through rates (41.7%, 250 times), discussions (33.3%, 200 times), and sharing (25%, 150 times), signifying substantial user interest in the related articles (Table 8 and Figure 8B).

**Table 7:** User comment sentiment analysis.

Affective category	Number of comments	Percent
Positive	120	40%
Neutral	105	35%
Passive	75	25%

**Table 8:** User behavior data analysis.

Behavior category	Frequency of action	Percent
Share	150	25%
Discuss	200	33.3%
Click rate	250	41.7%

### Multidimensional assessment of SSc information quality

**Figure 9:**
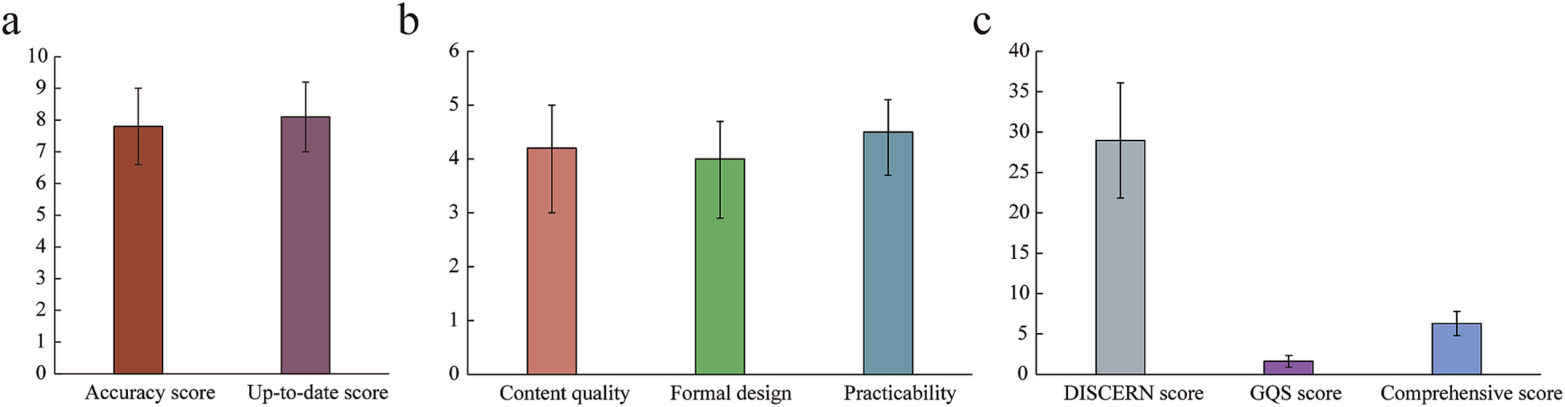
Multidimensional assessment results. **(a)** Accuracy and currency ratings of information. **(b)** Reader satisfaction survey results. **(c)** Composite evaluation index system rating.

For a comprehensive and accurate evaluation of treatment-related information on the WeChat platform concerning SSc, this study introduced various tools for assessing the quality of medical information. Firstly, accuracy and timeliness assessments were conducted by experts in the rheumatology field to rate the content of the articles, ensuring the accuracy and timeliness of the information. The expert ratings showed an average accuracy score of 7.8 (SD = 1.2), and an average timeliness score of 8.1 (SD = 1.1) (Figure 9a). A reader satisfaction survey was also designed and distributed to collect readers’ feedback on the articles’ content, format, and utility through the WeChat public platform. The survey results were compared and analyzed alongside scores from other evaluation tools to provide a comprehensive quality assessment. The reader satisfaction survey indicated an average score of 4.2 for content quality (SD = 0.8), a 4.0 average score for design (SD = 0.7), and a 4.5 average score for utility (SD = 0.6), suggesting overall high satisfaction (Figure 9b). Finally, by combining DISCERN and GQS scores, a comprehensive evaluation index system was developed to rate and rank the overall quality of the articles. The results of the comprehensive evaluation index system demonstrated that the average DISCERN score was 28.96 (SD = 7.13), the average GQS score was 1.62 (SD = 0.72), and the average composite score was 6.3 (SD = 1.5) (Figure 9c).

## Discussion

This study aimed to systematically evaluate the quality of treatment-related information on SSc published via WeChat and to explore factors influencing the establishment and long-term operation of rare disease public accounts. A total of 375 treatment-related articles were randomly selected from various SSc-related WeChat accounts. Their sources, content, and dissemination characteristics were analyzed. The quality of the articles was assessed using the DISCERN and GQS tools, and interaction metrics such as comments, shares, and views were extracted via the WeChat platform interface to examine the relationship between information quality and user engagement. Results showed that although most articles were published by medical non-profit organizations and focused on health education, the overall quality was generally low. High-quality accounts were limited, making it difficult to fully meet the information needs of patients. This study provides data support for evaluating the quality of rare disease-related health content on WeChat and offers guidance for improving communication strategies and enhancing public access to reliable medical information.

Low-quality health information may pose multiple risks to patients with SSc. Misleading content can lead to delayed recognition of early symptoms such as Raynaud’s phenomenon or skin thickening, resulting in missed opportunities for timely intervention. Some online articles underestimate the severity of SSc, mischaracterizing it as a minor skin or circulatory issue and delaying professional care. Claims about herbal remedies or supplements “curing” SSc, commonly found on social media, often lack scientific support and may cause side effects or interfere with standard treatments (46, 47). On platforms like WeChat, traditional medicine content emphasizing “natural and side-effect-free” remedies may mislead patients into avoiding evidence-based treatments. Exaggerated descriptions of disease severity may also increase patient anxiety and affect mental health. Exposure to information that contradicts medical advice can weaken trust in healthcare professionals and reduce treatment adherence. Improving the quality of SSc-related health information is therefore critical to support informed decision-making, timely care, and long-term disease management. Articles authored by non-profit organizations demonstrated higher quality and broader reach. The significant variation in content quality across WeChat highlights the need for improved health communication strategies. Using tools such as DISCERN and GQS strengthens the reliability of the findings and provides a scientific foundation for enhancing the quality of SSc-related content on social media platforms.

This study identified several key similarities and differences compared to previous research on social media health information dissemination. Whereas previous studies often regarded health information authored by companies as high in quality and credibility, this study found that articles written by companies had relatively lower quality, possibly due to the authors’ professional backgrounds and review mechanisms within these companies. Non-profit organizations, on the other hand, rigorously vet their information before publication, often having it written by professionals, which may be a primary reason for the higher quality of their articles (48–50). Articles written by individuals varied in quality, reflecting gaps in their medical knowledge and writing standards (51).

The study revealed a correlation between information quality and reader engagement. High-quality articles tended to attract more views, likes, and comments, aligning with findings on other social media platforms. Specifically, articles on policy interpretations and functional rehabilitation addressed readers’ practical needs and demonstrated higher engagement levels (52, 53). Improving information quality enhances reader engagement and fosters trust in the information source (54–56).

In this study, the quality of health information was systematically assessed using two evaluation tools, DISCERN and GQS. DISCERN primarily evaluates the reliability and quality of treatment choices in information, while GQS assesses the overall quality and utility of the information (57). The results indicated that most WeChat articles received low DISCERN and GQS scores, indicating subpar quality. Compared to other information quality assessment tools, DISCERN and GQS are straightforward and provide comprehensive evaluations; however, they still entail subjectivity. Future research could integrate multiple evaluation tools to enhance the objectivity and accuracy of assessment results (58). These strategies will enhance the effectiveness of systemic sclerosis information dissemination on WeChat, better meeting users’ health information needs.

WeChat faces challenges in disseminating information about systemic sclerosis, including varying credibility and content quality, but it also offers unique opportunities, such as expert Q&A and interaction in the comment section (59, 60). Enhancing the professionalism and training of information publishers can improve content quality (61). Non-profit organizations are crucial in spreading high-quality information that positively impacts patient education and disease management. Standardizing content review and publishing processes can help raise the overall quality of health information on the platform.

Improving the quality and efficiency of WeChat health communication requires establishing a trusted information environment that offers better support and community connections for SSc patients. WeChat can prioritize and promote high-quality SSc articles based on metrics such as views, likes, and comments, enhancing reliable health information delivery. A multi-tiered content review system should be established to ensure that health information is professionally vetted, improving its scientific accuracy and reliability. This approach will not only increase the credibility of information sources but also motivate authors to produce higher-quality content. Encouraging patients and families to share personal stories or treatment experiences and integrating them into the content will foster engagement and provide valuable insights for others.

Operating a WeChat public account dedicated to RDs requires a focus on professionalism, interactivity, and sustainability (23). Non-profit organizations have accumulated valuable experience through long-term operations, and their successful practices can serve as models for other public accounts (50, 62, 63). For instance, strategies such as regularly publishing high-quality articles, organizing online and offline activities, and providing professional consultation services (64–66) are crucial. Reader engagement is also a key operational strategy; enhancing user loyalty and trust by responding to reader comments and inquiries is essential. In the future, public accounts should pay further attention to the content’s professional nature and readers’ actual needs in information dissemination (67, 68).

This study has several limitations. First, the sample was drawn from only nine WeChat public accounts, which may not fully represent all SSc-related health information on the platform, introducing potential selection bias. Second, although DISCERN and GQS are evidence-based tools, the evaluation process is still influenced by subjective judgment. Differences in interpreting criteria such as “reliability” or “completeness” may lead to inconsistent scores. Moreover, the GQS provides a relatively general assessment, making it less effective in distinguishing finer differences in content quality.

Future research can improve objectivity and reliability by increasing the number of evaluators and conducting inter-rater reliability analyses, such as calculating Cronbach’s alpha or ICC. NLP and other AI tools are also recommended to evaluate scientific accuracy and content completeness, reducing human bias. Incorporating objective indicators, such as citation of authoritative sources, data support, and frequency of updates, may enhance comprehensiveness. Engaging patients and experts in the evaluation process can combine user experience with professional judgment, improving practical relevance. The Delphi method could be used to develop more detailed and consistent scoring criteria. Future work should also examine long-term dissemination metrics such as reader feedback, link clicks, and social engagement. Expanding the sample and comparing it across different social media platforms will help to better understand the role of WeChat in health information dissemination.

Overall, this study found that the general quality of SSc-related health information on WeChat is low, though content from non-profit organizations showed better quality and wider reach. The findings provide scientific and clinical references for improving the quality and effectiveness of rare disease communication on the platform. To enhance content quality, the implementation of expert review labels, source credibility tags, and classification systems is suggested. Introducing user rating mechanisms may increase transparency, interaction, and trust, supporting patient education and disease management. These limitations highlight the need for larger-scale studies to validate and extend the current findings. Improving publisher training, content accuracy, and user engagement may better serve patients and families affected by SSc, ultimately improving their quality of life and disease-coping capacity.

## Conclusion

This study highlights WeChat public accounts as an important channel for disseminating information on systemic sclerosis (SSc), with non-profit organizations contributing the most reliable content. While disease education dominates published materials, users are more interested in policy and rehabilitation topics. Overall, the quality of SSc-related information is low, especially in content authored by individuals or commercial entities, as reflected by DISCERN and GQS assessments.

Despite high readership, user engagement remains limited, suggesting a need for better interaction mechanisms. Expert evaluations indicate acceptable accuracy and timeliness, but further improvement is needed in treatment-related content and quality of life discussions. Reader satisfaction was generally high, though feedback suggests enhancing content design and practicality.

**Figure 10:**
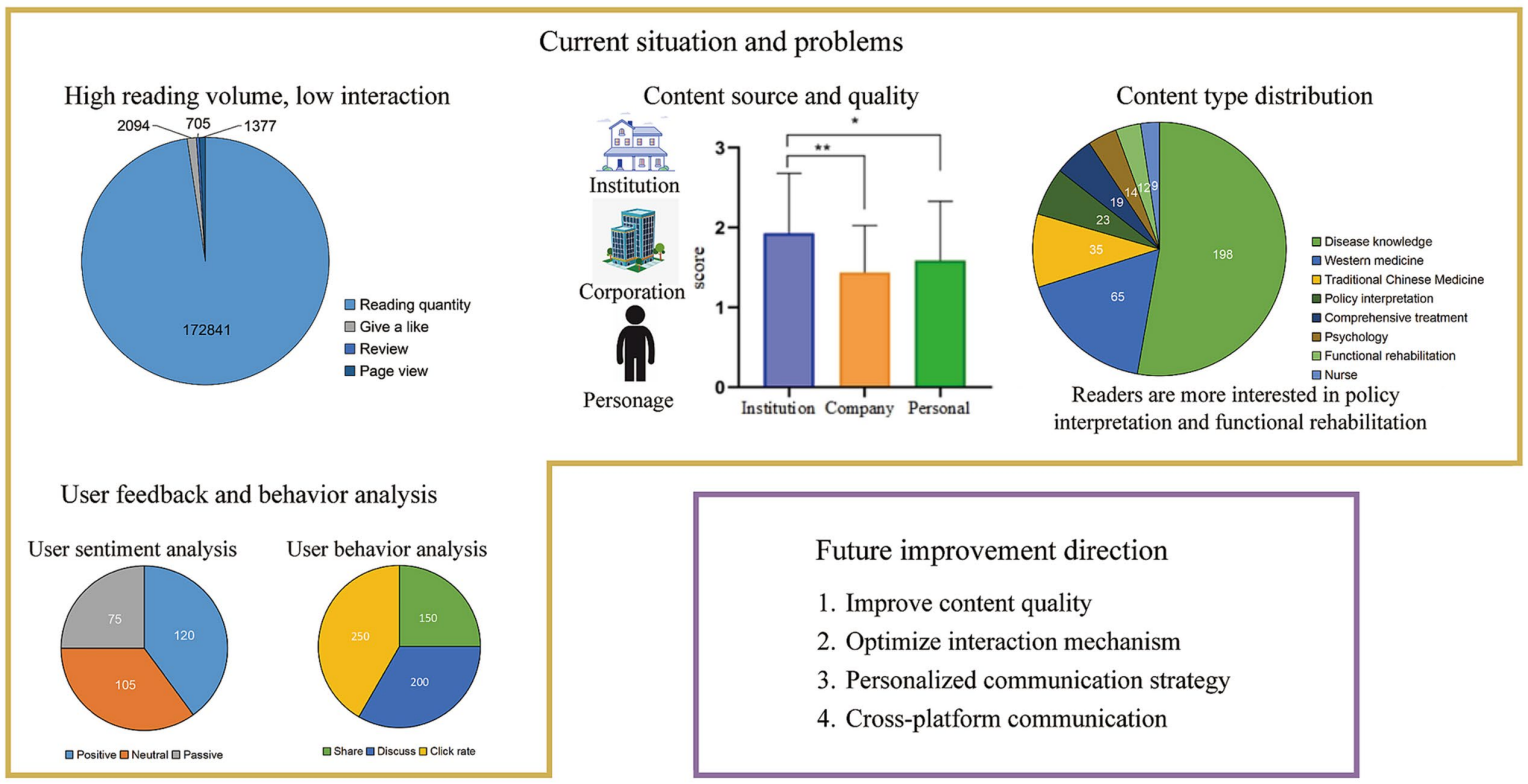
Current situation and improvement direction of SSc information dissemination on WeChat platform.

This study underscores the importance of evaluating health information quality on social media. Clinically, it supports the need to guide patients toward trustworthy sources to improve decision-making, treatment adherence, and outcomes (Figure 10).

## Data Availability

The original contributions presented in the study are included in the article/supplementary material, further inquiries can be directed to the corresponding author.

## References

[1] Rosendahl A Schönborn K Krieg T. Pathophysiology of systemic sclerosis (scleroderma). Kaohsiung J Med Scie. (2022) 38:187–95. doi: 10.1002/kjm2.12505, PMID: 35234358 PMC11896191

[2] Bukiri H Volkmann ER. Current advances in the treatment of systemic sclerosis. Curr Opin Pharmacol. (2022) 64:102211. doi: 10.1016/j.coph.2022.102211, PMID: 35447517 PMC9466985

[3] Lepri G Orlandi M Di Battista M De Mattia G Da Rio M Codullo V. Systemic sclerosis: one year in review 2022. Clin Exp Rheumatol. (2022). 40:1911–1920 doi: 10.55563/clinexprheumatol/3401fl, PMID: 36135958

[4] Gagarine M Cañedo-Ayala M Cook V Discepola M-N Guillot G Leader DM. Oral health in systemic sclerosis: a scoping review. J Scleroderma Relat Disord. (2024) 10:13–26. doi: 10.1177/23971983241252899, PMID: 39552945 PMC11561949

[5] Furst DE Fernandes AW Iorga ŞR Greth W Bancroft T. Epidemiology of systemic sclerosis in a large US managed care population. J Rheumatol. (2012) 39:784–6. doi: 10.3899/jrheum.111106, PMID: 22382343

[6] Ciaffi J Morabito MF Ruscitti P D’Angelo S Mancarella L Brusi V. Incidence, prevalence and mortality of systemic sclerosis in Italy: a nationwide population-based study using administrative health data. Rheumatol Int. (2020) 41:129–37. doi: 10.1007/s00296-020-04720-3, PMID: 33052445

[7] Gabrielli A Avvedimento EV Krieg T. Scleroderma. N Engl J Med. (2009) 360:1989–2003. doi: 10.1056/NEJMra0806188, PMID: 19420368

[8] Salvador CD Keith BA Ward C Nguyen SA Gordis T Chidarala S. Audiovestibular symptoms in systemic sclerosis: a systematic review and meta-analysis. Eur Arch Otorrinolaringol. (2024) 282:1147–57. doi: 10.1007/s00405-024-09001-4, PMID: 39394332 PMC11890250

[9] Donkó Á Sharapova SO Kabat J Ganesan S Hauck FH Bergerson JRE. Clinical and functional spectrum of RAC2-related immunodeficiency. Blood. (2024) 143:1476–87. doi: 10.1182/blood.2023022098, PMID: 38194689 PMC11033590

[10] Pérez-Milena A Ramos-Ruiz JA Zafra-Ramirez N Noguera-Cuenca C Rodríguez-Bayón A Ruiz-Díaz B. Qualitative study on the use of emergency services by people with serious mental disorder in Spain. BMC Prim Care. (2023) 24:24. doi: 10.1186/s12875-023-02078-6, PMID: 37340353 PMC10280892

[11] Li Y Xiao L. Research on public health crisis early warning system based on context awareness. THC. (2022) 30:303–14. doi: 10.3233/THC-THC228029, PMID: 35124607 PMC9028670

[12] Pereira H Fehér G Tibold A Esgalhado G Costa V Monteiro S. The impact of internet addiction and job satisfaction on mental health symptoms among a sample of Portuguese workers. IJERPH. (2021) 18:6943. doi: 10.3390/ijerph18136943, PMID: 34209522 PMC8297207

[13] Khunte A Sangha V Oikonomou EK Dhingra LS Aminorroaya A Coppi A. Automated Diagnostic Reports from Images of Electrocardiograms at the Point-of-Care. (2024). [Epubh ahead of preprint]. doi: 10.1101/2024.02.17.24302976, PMID: 38405776 PMC10889032

[14] Oikonomou EK Vaid A Holste G Coppi A McNamara RL Baloescu C. (2024). Artificial intelligence-guided detection of under-recognized cardiomyopathies on point-of-care cardiac ultrasound: a multi-center study doi: 10.1101/2024.03.10.24304044 [Epubh ahead of preprint]. PMC12084816 39890242

[15] Wang L Situ S Rainey JJ He B Su X Moolenaar RL. Using Weibo and WeChat social media channels to assess public awareness and practices related to antimicrobial resistance, China, 2019. BMC Public Health. (2021) 21:921. doi: 10.1186/s12889-021-10648-5, PMID: 33990188 PMC8120725

[16] Pan X Luo Y. Exploring the multidimensional relationships between social media support, social confidence, perceived media credibility and life attitude during the COVID-19 pandemic. Curr Psychol. (2022) 41:3388–400. doi: 10.1007/s12144-022-03238-0, PMID: 35645549 PMC9130692

[17] Yin M Chen S Pan X Lu C Lin X Wang M. Effects of Chinese provincial CDCs WeChat official account article features on user engagement during the COVID-19 pandemic. J Glob Health. (2023) 13:13. doi: 10.7189/jogh.13.06005, PMID: 37054436 PMC10101727

[18] Li Y Xiao Q-L Li M Zhang Y Chen M Jiang C-H. Community-based intervention via WeChat official account to improve parental health literacy among primary caregivers of children aged 0 to 3 years: protocol for a cluster randomized controlled trial. Front Public Health. (2023) 10:1039394. doi: 10.3389/fpubh.2022.1039394, PMID: 36684867 PMC9853903

[19] Liang X Yan M Li H Deng Z Lu Y Lu P. WeChat official accounts’ posts on medication use of 251 community healthcare centers in Shanghai, China: content analysis and quality assessment. Front Med. (2023) 10:1155428. doi: 10.3389/fmed.2023.1155428, PMID: 37378298 PMC10291264

[20] Fan Y Wang Z Deng S Lv H Wang F. The function and quality of individual epidemic prevention and control apps during the COVID-19 pandemic: a systematic review of Chinese apps. Int J Med Inform. (2022) 160:104694. doi: 10.1016/j.ijmedinf.2022.104694, PMID: 35144100 PMC8801898

[21] Jiang M Wang Q Finch T She D Zhou Y Chung YF. Validity and reliability of the Chinese version of the normalization MeAsure development(NoMAD). BMC Health Serv Res. (2022) 22:1338. doi: 10.1186/s12913-022-08737-1, PMID: 36368997 PMC9652982

[22] Yang M Suanpong K Ruangkanjanases A Yu W Xu H. Development and validity test of social attachment multidimensional scale. Front Psychol. (2022) 12:12. doi: 10.3389/fpsyg.2021.757777, PMID: 35058839 PMC8763980

[23] Liu LL Lai XF Xu L Feng JN He BJ Zou SY. A cross-sectional study on current status of rare disease related health information based on WeChat official accounts in China. Zhonghua Liu Xing Bing Xue Za Zhi. (2020) 41:446–51. doi: 10.3760/cma.j.issn.0254-6450.2020.03.031 32294851

[24] Su JJ Yu DS. Effects of a nurse-led eHealth cardiac rehabilitation programme on health outcomes of patients with coronary heart disease: a randomised controlled trial. Int J Nurs Stud. (2021) 122:104040. doi: 10.1016/j.ijnurstu.2021.104040, PMID: 34333211

[25] Yeung AWK Tosevska A Klager E Eibensteiner F Tsagkaris C Parvanov ED. Medical and health-related misinformation on social media: bibliometric study of the scientific literature. J Med Internet Res. (2022) 24:e28152. doi: 10.2196/28152, PMID: 34951864 PMC8793917

[26] Buonerba C Calabrese AN Imperioso G Piscosquito A Verde A Vaia A. Instant messaging in Cancer care. Oncology. (2024) 102:659–62. doi: 10.1159/000536345, PMID: 38262385

[27] He H Xu J Sun M Shao J Deng X Zeng L. WeChat app combined CBL in oral medicine clinical training: a review. Medicine. (2023) 102:e33102. doi: 10.1097/MD.0000000000033102, PMID: 36930092 PMC10019247

[28] Chen X Zhou X Li H Li J Jiang H. The value of WeChat application in chronic diseases management in China. Comput Methods Prog Biomed. (2020) 196:105710. doi: 10.1016/j.cmpb.2020.105710, PMID: 32858284

[29] Chen J Gao B Wang K Lei Y Zhang S Jin S. WeChat as a platform for blending problem/case-based learning and paper review methods in undergraduate paediatric orthopaedics internships: a feasibility and effectiveness study. BMC Med Educ. (2023) 23:4269. doi: 10.1186/s12909-023-04269-2, PMID: 37158869 PMC10166020

[30] Cunich M Barakat-Johnson M Lai M Arora S Church J Basjarahil S. The costs, health outcomes and cost-effectiveness of interventions for the prevention and treatment of incontinence-associated dermatitis: a systematic review. Int J Nurs Stud. (2022) 129:104216. doi: 10.1016/j.ijnurstu.2022.104216, PMID: 35364428

[31] Josfeld L Huebner J. Development and analysis of quality assessment tools for different types of patient information – websites, decision aids, question prompt lists, and videos. BMC Med Inform Decis Mak. (2023) 23:111. doi: 10.1186/s12911-023-02204-5, PMID: 37344772 PMC10283325

[32] Uzun O. Assessment of reliability and quality of videos on medial epicondylitis shared on YouTube. Cureus. (2023) 15:e37250. doi: 10.7759/cureus.37250, PMID: 37168186 PMC10166569

[33] Esen Özdemir E Borman P Mete Civelek G Umaroğlu MM. YouTube as a source of information on lipedema: property, quality, and reliability assessment. Lymphat Res Biol. (2023) 21:403–9. doi: 10.1089/lrb.2022.0028, PMID: 36927077

[34] Li B Liu M Liu J Zhang Y Yang W Xie L. Quality assessment of health science-related short videos on TikTok: a scoping review. Int J Med Inform. (2024) 186:105426. doi: 10.1016/j.ijmedinf.2024.105426, PMID: 38531256

[35] Uz C Umay E Ballı Uz F Cankurtaran D Gundogdu I. Assessment of the quality and reliability of the information on spasticity on youtube. J Clin Neurosci. (2023) 113:142–6. doi: 10.1016/j.jocn.2023.05.018, PMID: 37269749

[36] Çetinkaya Yaprak A Erkan PÇ. Assessment of the quality of information on treatment of keratoconus on YouTube. Int Ophthalmol. (2021) 42:1499–505. doi: 10.1007/s10792-021-02139-8, PMID: 34845598

[37] Ertekin SC. An assessment of the quality and reliability of gastric Botox information videos on YouTube. Cureus. (2023) 15:e44747. doi: 10.7759/cureus.44747, PMID: 37809228 PMC10556258

[38] Tian T Zhang J Xie W Ni Y Fang X Liu M. Dietary quality and relationships with metabolic dysfunction-associated fatty liver disease (MAFLD) among United States adults, results from NHANES 2017–2018. Nutrients. (2022) 14:4505. doi: 10.3390/nu14214505, PMID: 36364767 PMC9659246

[39] Pattenden TA Raleigh RA Pattenden ER Thangasamy IA. Quality and readability of online patient information on treatment for erectile dysfunction. BJUI Compass. (2021) 2:412–8. doi: 10.1002/bco2.87, PMID: 35474701 PMC8988690

[40] Zhan Z Su Z-W Chang H-L. Education and quality of life: does the internet matter in China? *Front*. Public Health. (2022) 10:297. doi: 10.3389/fpubh.2022.860297, PMID: 35372198 PMC8971524

[41] Sun F Zheng S Wu J. Quality of information in gallstone disease videos on TikTok: cross-sectional study. J Med Internet Res. (2023) 25:e39162. doi: 10.2196/39162, PMID: 36753307 PMC9947761

[42] Ved R Cobbold N Igbagiri K Willis M Leach P Zaben M. Online patient information on Vagus nerve stimulation: how reliable is it for facilitating shared decision making? Seizure. (2017) 50:125–9. doi: 10.1016/j.seizure.2017.06.009, PMID: 28651125

[43] Zhang X Yang Y Shen Y-W Zhang K-R Ma L-T Ding C. Quality of online video resources concerning patient education for neck pain: a YouTube-based quality-control study. Front Public Health. (2022) 10:972348. doi: 10.3389/fpubh.2022.972348, PMID: 36211682 PMC9533122

[44] Kocyigit BF Nacitarhan V Koca TT Berk E. YouTube as a source of patient information for ankylosing spondylitis exercises. Clin Rheumatol. (2019) 38:1747–51. doi: 10.1007/s10067-018-04413-0, PMID: 30645752

[45] Singh AG Singh S Singh PP. YouTube for information on rheumatoid arthritis — a wakeup call? J Rheumatol. (2012) 39:899–903. doi: 10.3899/jrheum.111114, PMID: 22467934

[46] Devgire V Martin AF McKenzie L Sandler RD Hughes M. A systematic review of internet-based information for individuals with Raynaud’s phenomenon and patients with systemic sclerosis. Clin Rheumatol. (2020) 39:2363–7. doi: 10.1007/s10067-020-05023-5, PMID: 32152916

[47] Yildirim TD Basibuyuk F Birlik AM. Patients with systemic sclerosis frequently use phytopharmaceuticals: a cross-sectional survey. Rheumatol Int. (2024) 44:3079–85. doi: 10.1007/s00296-024-05745-8, PMID: 39466382

[48] Wirth T Peters C Nienhaus A Schablon A. Interventions for workplace violence prevention in emergency departments: a systematic review. IJERPH. (2021) 18:8459. doi: 10.3390/ijerph18168459, PMID: 34444208 PMC8392011

[49] Nakajima M Yamada S Miyajima M Ishii K Kuriyama N Kazui H. Guidelines for Management of Idiopathic Normal Pressure Hydrocephalus (third edition): endorsed by the Japanese Society of Normal Pressure Hydrocephalus. Neurol Med Chir. (2021) 61:63–97. doi: 10.2176/nmc.st.2020-0292 PMC7905302 33455998

[50] Haeuser E Serfes AL Cork MA Yang M Abbastabar H Abhilash ES. Mapping age- and sex-specific HIV prevalence in adults in sub-Saharan Africa, 2000–2018. BMC Med. (2022) 20:488. doi: 10.1186/s12916-022-02639-z, PMID: 36529768 PMC9760541

[51] Díaz-Guio DA Díaz-Guio Y Pinzón-Rodas V Díaz-Gomez AS Guarín-Medina JA Chaparro-Zúñiga Y. COVID-19: biosafety in the intensive care unit. Curr Trop Med Rep. (2020) 7:104–11. doi: 10.1007/s40475-020-00208-z, PMID: 32868986 PMC7449784

[52] Shiraiwa M Fang T Wei J Lakey P Hwang B Edwards KC. Chemical and cellular formation of reactive oxygen species from secondary organic aerosols in epithelial lining fluid. Res Rep Health Eff Inst. (2023) 2023:1–56. PMID: 38420854 PMC10957138

[53] Dunne KS Holden NM Daly K. A management framework for phosphorus use on agricultural soils using sorption criteria and soil test P. J Environ Manag. (2021) 299:113665. doi: 10.1016/j.jenvman.2021.113665, PMID: 34479156

[54] Suarez-Lledo V Alvarez-Galvez J. Prevalence of health misinformation on social media: systematic review. J Med Internet Res. (2021) 23:e17187. doi: 10.2196/17187, PMID: 33470931 PMC7857950

[55] Dolan SB Mahon BE Dowell SF Zaidi A. Toward understanding death. Clin Infect Dis. (2021) 73:S341–2. doi: 10.1093/cid/ciab809, PMID: 34910167 PMC8672748

[56] Lee S-Y Lee S. Quality of online information regarding high-risk pregnancies. Comput Inform Nurs. (2021) 39:974–83. doi: 10.1097/CIN.0000000000000768 34145205

[57] Dembinski D Moore MG Effendi M Ovalle F Gobble R. Assessing the quality and reliability of patient information regarding aesthetic fat grafting on YouTube. J Plast Reconstr Aesthet Surg. (2022) 75:2343–5. doi: 10.1016/j.bjps.2022.01.053, PMID: 35272959

[58] Heisinger S Huber D Matzner MP Hiertz H Lampe LP Zagata J. TLIF online videos for patient education—evaluation of comprehensiveness, quality, and reliability. IJERPH. (2023) 20:4626. doi: 10.3390/ijerph20054626, PMID: 36901636 PMC10002268

[59] Guo S Kaminga AC Xiong J. Depression and coping styles of college students in China during COVID-19 pandemic: a systemic review and Meta-analysis. Front Public Health. (2021) 9:9. doi: 10.3389/fpubh.2021.613321, PMID: 34307268 PMC8292621

[60] Ma X Lu J Liu W. Influencing factors on health information to improve public health literacy in the official WeChat account of Guangzhou CDC. Front Public Health. (2021) 9:9. doi: 10.3389/fpubh.2021.657082, PMID: 34414152 PMC8369197

[61] Ni X Lou Y Hu W Wang H Xu H Li S. Development of mobile health–based self-management support for patients with lung cancer: a stepwise approach. Nurs Open. (2022) 9:1612–24. doi: 10.1002/nop2.1185, PMID: 35128821 PMC8994947

[62] Trubetskoy V Pardiñas AF Qi T Panagiotaropoulou G Awasthi S Bigdeli TB. Mapping genomic loci implicates genes and synaptic biology in schizophrenia. Nature. (2022) 604:502–8. doi: 10.1038/s41586-022-04434-5, PMID: 35396580 PMC9392466

[63] Tromp TR Hartgers ML Hovingh GK Vallejo-Vaz AJ Ray KK Soran H. Worldwide experience of homozygous familial hypercholesterolaemia: retrospective cohort study. Lancet. (2022) 399:719–28. doi: 10.1016/S0140-6736(21)02001-8, PMID: 35101175 PMC10544712

[64] Kars ME Başak AN Onat OE Bilguvar K Choi J Itan Y. The genetic structure of the Turkish population reveals high levels of variation and admixture. Proc Natl Acad Sci USA. (2021) 118:6118. doi: 10.1073/pnas.2026076118, PMID: 34426522 PMC8433500

[65] Wang Y Fan JL Melms JC Amin AD Georgis Y Barrera I. Multimodal single-cell and whole-genome sequencing of small, frozen clinical specimens. Nat Genet. (2023) 55:19–25. doi: 10.1038/s41588-022-01268-9, PMID: 36624340 PMC10155259

[66] Wang C Dai J Qin N Fan J Ma H Chen C. Analyses of rare predisposing variants of lung cancer in 6,004 whole genomes in Chinese. Cancer Cell. (2022) 40:1223–1239.e6. doi: 10.1016/j.ccell.2022.08.013, PMID: 36113475

[67] Smith JS Seddighi R. Miniature companion pig sedation and anesthesia. Vet Clin North Am Exot Anim Pract. (2022) 25:297–319. doi: 10.1016/j.cvex.2021.08.007, PMID: 34823695

[68] McNary AL. Maintenance and destruction of treatment records. Innov Clin Neurosci. (2021) 18:44–7. PMID: 34980984 PMC8667710

